# The Shared Mechanism and Candidate Drugs of Multiple Sclerosis and Sjögren’s Syndrome Analyzed by Bioinformatics Based on GWAS and Transcriptome Data

**DOI:** 10.3389/fimmu.2022.857014

**Published:** 2022-03-09

**Authors:** Xiangxiang Hong, Xin Wang, Xinming Rang, Xinyue Yin, Xuemei Zhang, Rui Wang, Duo Wang, Tingting Zhao, Jin Fu

**Affiliations:** ^1^ Department of Neurology, The Second Affiliated Hospital of Harbin Medical University, Harbin, China; ^2^ Department of Neurology, The First Affiliated Hospital of Harbin Medical University, Harbin, China

**Keywords:** multiple sclerosis, Sjögren’s syndrome, GWAS, transcriptome, comorbidity

## Abstract

**Objective:**

This study aimed to explore the shared mechanism and candidate drugs of multiple sclerosis (MS) and Sjögren’s syndrome (SS).

**Methods:**

MS- and SS-related susceptibility genes and differentially expressed genes (DEGs) were identified by bioinformatics analysis based on genome-wide association studies (GWAS) and transcriptome data from GWAS catalog and Gene Expression Omnibus (GEO) database. Pathway enrichment, Gene Ontology (GO) analysis, and protein–protein interaction analysis for susceptibility genes and DEGs were performed. The drugs targeting common pathways/genes were obtained through Comparative Toxicogenomics Database (CTD), DrugBank database, and Drug–Gene Interaction (DGI) Database. The target genes of approved/investigational drugs for MS and SS were obtained through DrugBank and compared with the common susceptibility genes.

**Results:**

Based on GWAS data, we found 14 hub common susceptibility genes (*HLA-DRB1*, *HLA-DRA*, *STAT3*, *JAK1*, *HLA-B*, *HLA-DQA1*, *HLA-DQA2*, *HLA-DQB1*, *HLA-DRB5*, *HLA-DPA1*, *HLA-DPB1*, *TYK2*, *IL2RA*, and *MAPK1*), with 8 drugs targeting two or more than two genes, and 28 common susceptibility pathways, with 15 drugs targeting three or more than three pathways. Based on transcriptome data, we found 3 hub common DEGs (*STAT1*, *GATA3*, *PIK3CA*) with 3 drugs and 10 common risk pathways with 435 drugs. “JAK-STAT signaling pathway” was included in common susceptibility pathways and common risk pathways at the same time. There were 133 overlaps including JAK-STAT inhibitors between agents from GWAS and transcriptome data. Besides, we found that *IL2RA* and *HLA-DRB1*, identified as hub common susceptibility genes, were the targets of daclizumab and glatiramer that were used for MS, indicating that daclizumab and glatiramer may be therapeutic for SS.

**Conclusion:**

We observed the shared mechanism of MS and SS, in which JAK-STAT signaling pathway played a vital role, which may be the genetic and molecular bases of comorbidity of MS with SS. Moreover, JAK-STAT inhibitors were potential therapies for MS and SS, especially for their comorbidity.

## 1 Introduction

Multiple sclerosis (MS) is a chronic inflammatory demyelinating disease of the central nervous system (CNS) (1). The etiology and pathogenesis of MS are complex, in which autoimmune responses based on genetic susceptibility and environmental risk factors have a vital role. However, the initiation of autoimmune responses is not fully explained, which may be related to Epstein–Barr virus (EBV) antigen mimicking or epitope expansion in genetically susceptible individuals (2).

Comorbidity of MS with many other autoimmune diseases is found in our clinical work, including Sjögren’s syndrome (SS). Epidemiological studies show that the prevalence of SS in MS patients is about 1% up to 16.7% in primary progressive MS patients, with the incidence of sicca syndrome (typical symptoms but not meeting the diagnostic criteria of SS) up to 10%, while the prevalence of SS in the general population is only 0.06%, indicating that the prevalence of SS in MS patients is higher than that in the general population (3). On the other hand, SS also involves CNS with a 5.8%–38% probability, and 52%–80% of these conditions occur before SS diagnosis (3).

SS is characterized by exocrinopathy and specific anti-SSA/SSB antibodies, resulting in dryness of the mouth and eyes, fatigue, and joint pain, can also involve CNS (4). The etiology and pathogenesis of SS are not clear. Both MS and SS are associated with the same environmental factors including EBV and cytomegalovirus (CMV) infection, vitamin D deficiency, and smoking (1, 5, 6). In addition, SS is also associated with autoimmune responses mainly mediated by T and B cells, which may be activated by abnormal mucosal epithelial cells stimulated by herpesvirus such as EBV (4). T cells play a major role in the early periods of SS, especially CD4^+^ T cells while B cells contribute more in later progression (5).

Based on the epidemiological correlation between MS and SS and the similarities in environmental factors and pathogenesis, we speculated that there may be common genetic susceptibility factors and pathogenic pathways and then explored whether the therapeutic agents of these two diseases can provide reference for each other. Therefore, based on the available genome-wide association studies (GWAS) and transcriptome data, we analyzed the shared genes and pathways between MS and SS from different levels of susceptibility genes and differentially expressed genes (DEGs) and explored potential therapeutic drugs. Our study may be helpful in revealing the shared genetic etiology and pathogenesis underlying MS and SS. More importantly, these findings may provide clues to potential therapeutic strategies.

## 2 Methods

### 2.1 Processing and Analysis of GWAS Data

#### 2.1.1 GWAS Data Extraction and Identification of Susceptibility Genes

GWAS is a database that systematically summarizes the observed single-nucleotide polymorphism (SNP)–disease associations. GWAS data of MS and SS were obtained and downloaded from the GWAS Catalog (https://www.ebi.ac.uk/gwas/) up to January 2021 through searching “multiple sclerosis” and “Sjögren’s syndrome,” respectively, which include disease-related SNPs, reported genes, PubMed ID, sample size, ethical groups, *P*-value, study accession ID, and platform (**Supplementary Table S1** and **Supplementary Figure S1**) (6). For SNPs reported in GWAS, those with *P*-value ≤5.0 × 10^–8^ were included in our study. And the corresponding reported genes were collected and defined as the susceptibility genes of MS or SS. Hypergeometric test was performed to evaluate the significance of overlap of MS and SS susceptibility genes: phyper (k-1, M, N-M, n, lower.tail=F).

#### 2.1.2 Enrichment Analysis and Functional Annotation of Common Susceptibility Genes

The Kyoto Encyclopedia of Genes and Genomes (KEGG) pathway enrichment analysis of the susceptibility genes was carried out by R package “clusterprofiler” (Version 4) (7, 8). Hypergeometric test was performed to evaluate the significance of pathway–pathway association: phyper (k-1, M, N-M, n, lower.tail=F). After common susceptibility pathways were obtained, susceptibility genes of MS/SS annotated to common susceptibility pathways were obtained and defined as common susceptibility genes in this study. And Gene Ontology (GO) annotation of the common susceptibility genes was performed by the Database for Annotation, Visualization and Integrated Discovery (DAVID) (version 6.8) (9, 10), including biological process (BP), cellular component (CC), and molecular function (MF). *P* **-**value <0.05 was determined as a significant margin for all analyses. Column chart was plotted by http://www.bioinformatics.com.cn, a free online platform for data analysis and visualization.

#### 2.1.3 Pathway–Gene Network Construction

The common susceptibility pathway–gene network was constructed through Cytoscape (version 3.8.2) (11), and the correlation degree of gene nodes was calculated.

#### 2.1.4 Protein–Protein Interaction Network Construction

The protein–protein interaction (PPI) network of common susceptibility genes was constructed using STRING (https://cn.string-db.org/, version 11.5) and visualized by Cytoscape, and the correlation degree of nodes was calculated (12).

### 2.2 Processing and Analysis of Transcriptome Data

#### 2.2.1 Transcriptome Data Extraction and Identification of Differentially Expressed Genes

Transcriptome data of MS and SS were obtained from the Gene Expression Omnibus (GEO) database (13). We evaluated two datasets with accession IDs GSE78244 and GSE117935 for MS and another two with GSE94510 and GSE135809 for SS. These four datasets are microarray gene expression data. GSE78244 dataset contains the data of CD4^+^ T cells from 14 MS and 14 healthy control subjects, while GSE117935 contains those of B cells from 10 MS and 10 healthy control subjects, GSE94510 contains those of CD4^+^ T cells from 6 SS and 6 healthy control subjects, and GSE135809 contains those of B cells from 6 SS and 6 healthy control subjects. The datasets were normalized by log2 transformation and quantile standardization using R package “Limma” (14). Significant DEGs with *P*-value <0.05 and fold change ≥1.2 were obtained by R, and common DEGs were identified by intersection.

#### 2.2.2 Enrichment Analysis and Functional Annotation of Common Differentially Expressed Genes

GO annotation of common DEGs was performed by DAVID (the same as above). The KEGG pathway enrichment analysis of the DEGs was carried out by R package “clusterprofiler” (the same as above). Significant risk pathways of MS and SS and common risk pathways were obtained.

#### 2.2.3 Protein–Protein Interaction Network Construction

The PPI network of common DEGs was constructed using STRING (the same as above).

### 2.3 Identification of Candidate Drugs for Multiple Sclerosis and Sjögren’s Syndrome

#### 2.3.1 Identification of Drugs Targeting Common Pathways/Genes

Drugs targeting common susceptibility pathways from GWAS data and common risk pathways from transcriptome data were identified using the Comparative Toxicogenomics Database (CTD) (version 16438) (15). Drugs targeting common susceptibility genes from GWAS data and common DEGs from transcriptome data were identified using DrugBank database (version 5.1.8) and Drug–Gene Interaction (DGI) Database (version 4.2.0) (16, 17). Then, drug–pathway and drug–gene interaction networks were constructed through Cytoscape. The drugs targeting three or more than three pathways and drugs targeting two or more than two genes were identified further. In addition, we compared the drugs identified from GWAS data and transcriptome data.

#### 2.3.2 Analysis of Target Genes of Approved/Investigational Drugs for Multiple Sclerosis and Sjögren’s Syndrome

Approved drugs and investigational drugs for MS and SS were searched with “multiple sclerosis” and “Sjögren’s syndrome” through the DrugBank database and *ClinicalTrials.gov* database (18). Investigational drugs were screened according to the following criteria. Inclusion criteria: ① The trial is in progress or has been completed; ② Definite drug composition. Exclusion criteria: ① Symptomatic drugs; ② Unclear composition; ③ Experiment terminated; ④ Non-drug treatment; ⑤ Approved drugs. Then, the target genes of drugs meeting the above criteria were obtained through the DrugBank database and compared with the common susceptibility genes found in this study.

**Figure 1:**
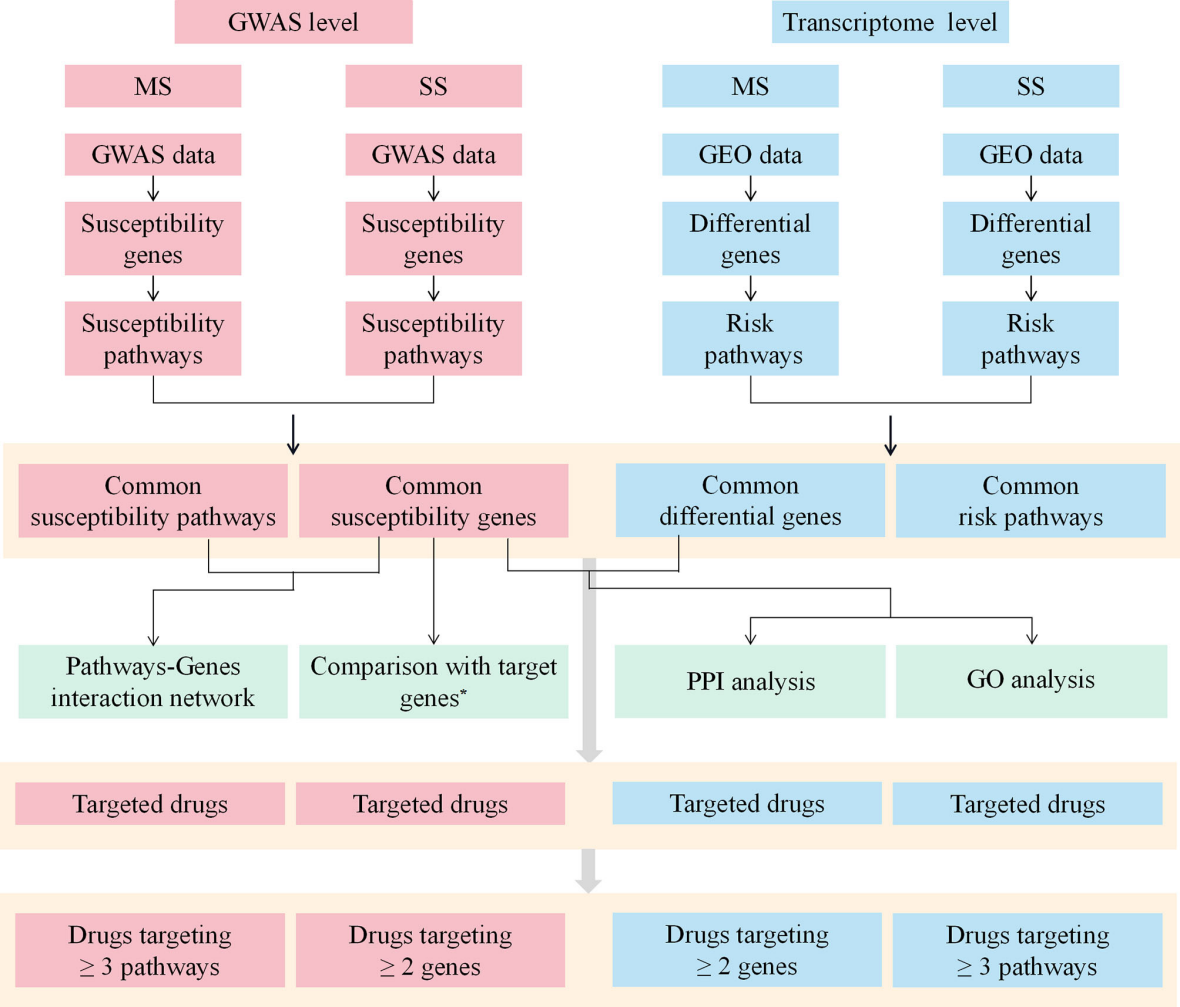
The workflow of the study. MS, multiple sclerosis; SS, sjögren's syndrome; GWAS, Genome-Wide Association Study; GEO, Gene Expression Omnibus; PPI, protein-protein interaction; GO, Gene ontology. *Target genes indicate the target genes of approved drugs and investigational drugs for MS and SS.

The workflow of this study was shown in **Figure 1**.

## 3 Results

### 3.1 Common Genes and Pathways of Multiple Sclerosis and Sjögren’s Syndrome Based on GWAS Data

#### 3.1.1 Identification of Susceptibility Genes and Enrichment Analysis

**Figure 2:**
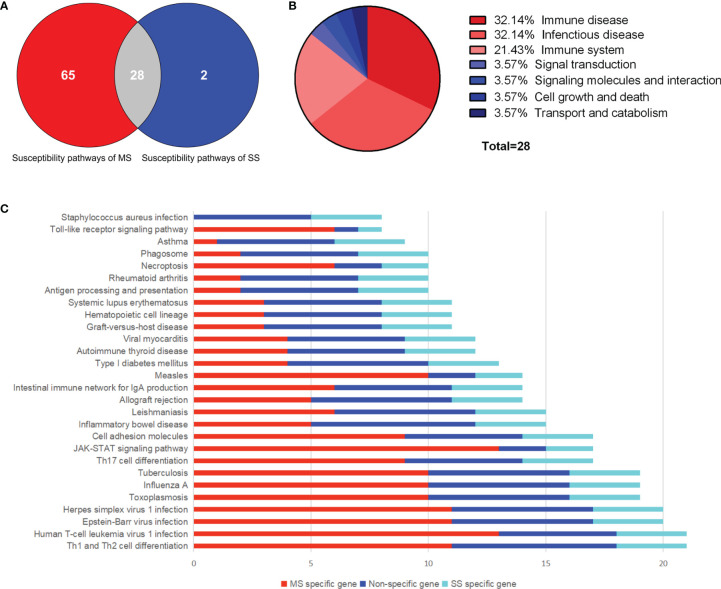
Common susceptibility pathways. **(A)** Venn diagram of common susceptibility pathways. **(B)** Classification of common susceptibility pathways. **(C)** Column chart of common susceptibility pathways. MS, multiple sclerosis; SS, sjögren's syndrome.

A total of 385 SNPs of MS and 19 SNPs of SS were obtained from GWAS data, as well as 260 and 22 susceptibility genes, respectively (**Supplementary Tables S1, S2** and **Supplementary Figures S1, S2**). Eleven overlapping genes with significance evaluated by hypergeometric test were found (*P* = 9.02 × 10^-16^). Ninety-three and 30 susceptibility pathways of MS and SS were obtained through KEGG pathway enrichment of susceptibility genes using R package “clusterprofiler” (*P* < 0.05). Twenty-eight common susceptibility pathways were obtained through Venn cross, and the classification was shown in **Figures 2A, B**. The first common susceptibility pathway according to the annotated gene number was the “Th1 and Th2 cell differentiation pathway” (**Figure 2C**). In order to evaluate the pathway–pathway association, the hypergeometric test was performed on 378 pathway–pathway pairs, of which 360 pairs were significant (*P* < 0.05) (**Supplementary Table S3**).

#### 3.1.2 Identification and Analysis of Common Susceptibility Genes

**Figure 3:**
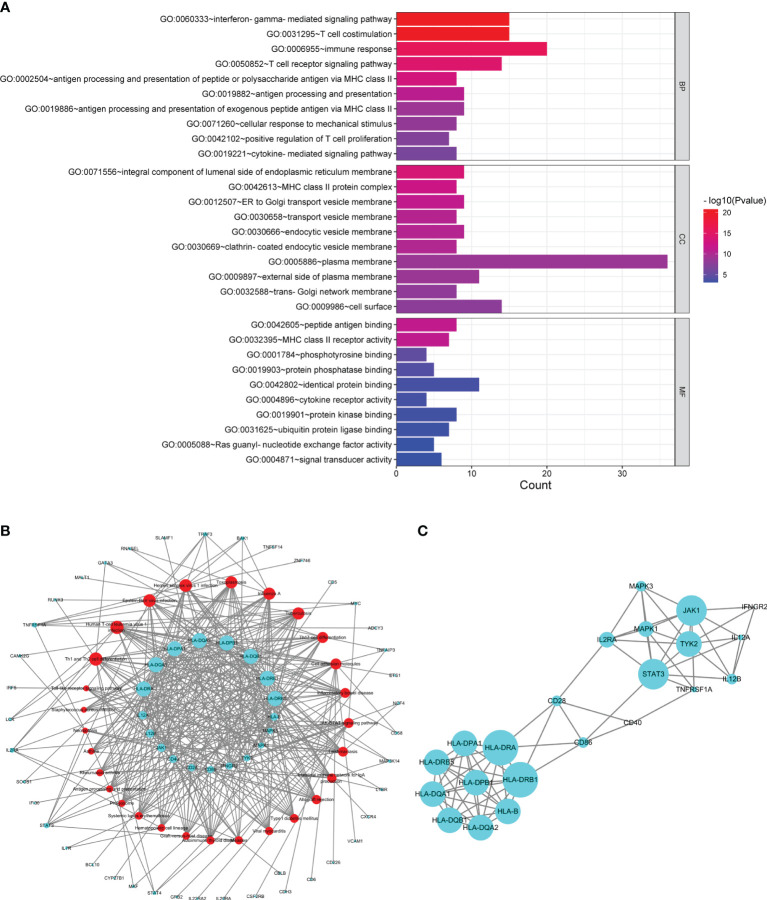
Analysis of common susceptibility genes. **(A)** Top 10 GO terms in the biological process, cellular component, and molecular function categories of common susceptibility genes. **(B)** Common susceptibility pathway–common susceptibility gene network. The red nodes represent pathways, and the green nodes represent genes. **(C)** Protein–protein interaction network of common susceptibility genes.

Sixty common susceptibility genes of MS and SS were obtained (**Supplementary Table S4**). A total of 189 enriched GO terms were identified for common susceptibility genes (**Supplementary Table S5**). GO annotation showed that the most significant BP was the “interferon-gamma-mediated signaling pathway”, CC was the “integral component of lumenal side of endoplasmic reticulum membrane”, and MF was the “peptide antigen binding”. The top 10 terms in the BP, CC, and MF categories were shown in **Figure 3A**.

From the common susceptibility pathway–gene network mapped by Cytoscape, 22 genes with degree ≥6 were identified (**Figure 3B**). Through PPI analysis and visualization by Cytoscape, 14 genes with degree ≥6 that were regarded as hub common susceptibility genes in our study (*HLA-DRB1*, *HLA-DRA*, *STAT3*, *JAK1*, *HLA-B*, *HLA-DQA1*, *HLA-DQA2*, *HLA-DQB1*, *HLA-DRB5*, *HLA-DPA1*, *HLA-DPB1*, *TYK2*, *IL2RA*, and *MAPK1*) were identified further (**Figure 3C**).

### 3.2 Common Genes and Pathways of Multiple Sclerosis and Sjögren’s Syndrome Based on Transcriptome Data

**Figure 4:**
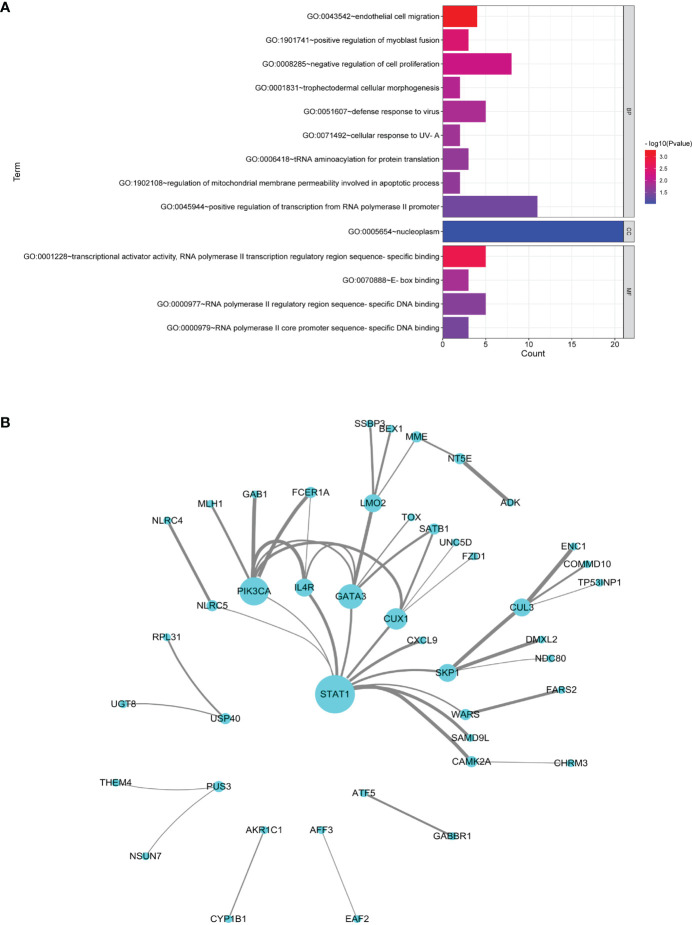
Analysis of common DEGs. **(A)** GO terms in the biological process, cellular component, and molecular function categories of common DEGs. **(B)** Protein–protein interaction network of common DEGs. Nodes represent DEGs, and edges represent the combined score of PPI. The higher the combined score, the thicker the edge.

MS and SS datasets were obtained from GEO database (**Table 1**). The list of DEGs, the fold change, and the associated *P*-values in CD4^+^ T and B cells in MS and SS were shown in **Supplementary Table S6**. There were 109 common DEGs of MS and SS, including 18 DEGs in CD4^+^ T cells and 91 DEGs in B cells. GO annotation showed that the most significant BP was “endothelial cell migration”; the CC with the most expressed genes was “nucleoplasm”, but it did not reach significance (*P* > 0.05), and the most significant MF was “transcriptional activator activity, RNA polymerase II transcription regulatory region sequence-specific binding” (**Figure 4A**). The PPI network with 45 nodes and 45 edges was constructed, and 3 hub common DEGs (*STAT1*, *PIK3CA*, and *GATA3*) with degree ≥6 were identified (**Figure 4B**). In terms of KEGG pathway enrichment, 35 and 74 significant risk pathways of MS and SS and 10 common risk pathways were obtained (**Table 2**).

**Table 1:** Characteristics of transcriptome datasets.

ID	Disease	Sample	Sample size (control)	Platform ID	Date	DEG	Pathway
GSE78244	MS	CD4^+^ T cell	28 (14)	GPL17077	2016	714	35
GSE117935	MS	B cell	20 (10)	GPL5175	2018	293	
GSE94510	SS	CD4^+^ T cell	12 (6)	GPL570	2017	2,514	74
GSE135809	SS	B cell	12 (6)	GPL570	2019	7,373	

**Table 2:** Common risk pathways of MS and SS.

KEGG_ID	KEGG_Term	*P*-value (MS)	*P*-value (SS)
hsa04630	JAK-STAT signaling pathway	0.019761431	0.002589011
hsa04614	Renin-angiotensin system	0.028086801	0.002850761
hsa04066	HIF-1 signaling pathway	0.001235913	0.003595271
hsa00380	Tryptophan metabolism	0.007088408	0.006280651
hsa00620	Pyruvate metabolism	0.047106391	0.007331886
hsa04061	Viral protein interaction with cytokine and cytokine receptor	0.008545436	0.007729449
hsa05164	Influenza A	0.027531183	0.014997505
hsa04550	Signaling pathways regulating pluripotency of stem cells	0.008786573	0.030629834
hsa04713	Circadian entrainment	0.007146748	0.032560706
hsa04728	Dopaminergic synapse	0.038600501	0.041333016

### 3.3 Candidate Drugs for Multiple Sclerosis and Sjögren’s Syndrome

#### 3.3.1 Drugs Targeting Common Susceptibility Pathways/Genes Based on GWAS Data

**Figure 5:**
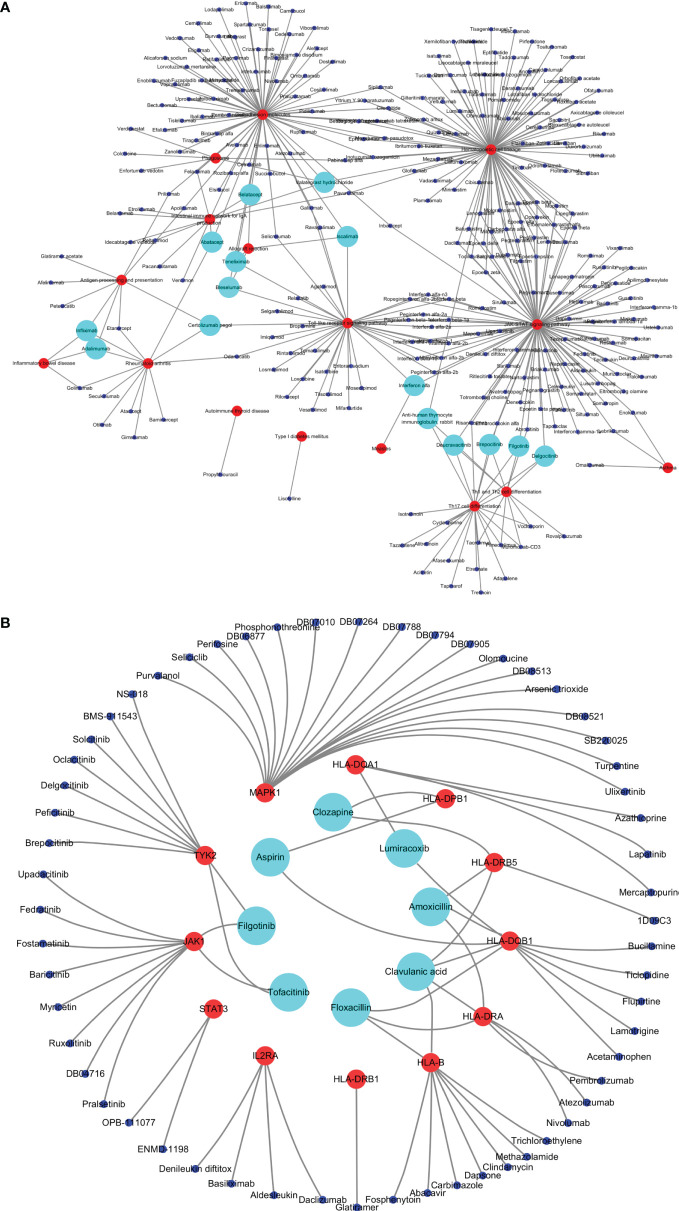
Drugs targeting common susceptibility pathways/genes. **(A)** Drugs–common susceptibility pathways interaction network. The red nodes represent pathways, the blue nodes represent drugs, and the green nodes represent drugs targeting more than or equal to three pathways. **(B)** Drugs–hub common susceptibility genes interaction network. The red nodes represent genes, the blue nodes represent drugs, and the green nodes represent drugs targeting more than or equal to two genes.

A total of 295 drugs targeting common susceptibility pathways were found using CTD, of which antineoplastic drugs accounted for 41%; anti-inflammatory drugs, 15%; and immunodepressant drugs, 5%. There were 15 drugs targeting more than or equal to three pathways, such as filgotinib, delgocitinib, and brepocitinib (**Figure 5A** and **Table 3**). In addition, 168 drugs targeting common susceptibility genes were obtained through the DrugBank and DGI databases, of which 22 drugs targeted more than or equal to two common susceptibility genes (**Table 4**). And there were 68 drugs targeting hub common susceptibility genes (**Figure 5B**), of which 8 drugs targeted more than or equal to two common susceptibility genes, for example, filgotinib and tofacitinib (**Table 4**).

**Table 3:** Drugs targeting more than or equal to three common susceptibility pathways.

Drug	Target pathway	Effect
Abatacept	Intestinal immune network for IgA production	Selective costimulation modulator
	Cell adhesion molecules	Antirheumatic
	Rheumatoid arthritis	
Adalimumab	Inflammatory bowel disease	Anti-TNF-α antibody
	Antigen processing and presentation	Antirheumatic
	Rheumatoid arthritis	Anti-inflammatory
Anti-human thymocyte	Th1 and Th2 cell differentiation	Immunosuppressant
Immunoglobulin (rabbit)	Th17 cell differentiation	
	Hematopoietic cell lineage	
Belatacept	Allograft rejection	Selective costimulation modulator
	Intestinal immune network for IgA production	Immunosuppressant
	Cell adhesion molecules	Antirheumatic
Bleselumab	Allograft rejection	Anti-CD40 antibody
	Cell adhesion molecules	Immunosuppressant
	Toll-like receptor signaling pathway	
Brepocitinib	Th1 and Th2 cell differentiation	JAK1, TYK2 dual inhibitor
	Th17 cell differentiation	Anti-inflammatory
	JAK-STAT signaling pathway	
Certolizumab pegol	Antigen processing and presentation	Anti-TNF-α antibody
	Rheumatoid arthritis	Anti-inflammatory
	Toll-like receptor signaling pathway	Antirheumatic
Delgocitinib	Th1 and Th2 cell differentiation	JAK inhibitor
	Th17 cell differentiation	Anti-inflammatory
	JAK-STAT signaling pathway	Antipsoriatic
Deucravacitinib	Th1 and Th2 cell differentiation	TYK2 inhibitor
	Th17 cell differentiation	Anti-inflammatory
	JAK-STAT signaling pathway	
Filgotinib	Th1 and Th2 cell differentiation	JAK inhibitor
	Th17 cell differentiation	Anti-inflammatory
	JAK-STAT signaling pathway	
Infliximab	Inflammatory bowel disease	Anti-TNF-α antibody
	Antigen processing and presentation	Anti-inflammatory
	Rheumatoid arthritis	Antirheumatic
Interferon alfa	JAK-STAT signaling pathway	Antineoplastic
	Measles	Antiviral
	Toll-like receptor signaling pathway	Biological response modifier
Iscalimab	Allograft rejection	Anti-CD40 antibody
	Cell adhesion molecules	
	Toll-like receptor signaling pathway	
Teneliximab	Allograft rejection	Anti-CD40 antibody
	Cell adhesion molecules	Immunosuppressant
	Toll-like receptor signaling pathway	
Valategrast	Intestinal immune network for IgA production	ITGA4/ITGB1, ITGA4/ITGB7 dual inhibitor
hydrochloride	Cell adhesion molecules	Antiasthmatic
	Hematopoietic cell lineage	
	Phagosome	

**Table 4:** Drugs targeting more than or equal to two common susceptibility genes. Black-bordered letters represent drugs targeting more than or equal to two hub common susceptibility genes.

Drug	Target gene	Effect
**Amoxicillin**	HLA-DQB1, HLA-DRA, HLA-DRB5	Antibacterial
**Aspirin**	HLA-DPB1, HLA-DQB1	NSAID
**Clavulanic acid**	HLA-B, HLA-DQB1, HLA-DRA, HLA-DRB5	β-lactamase inhibitor
**Clozapine**	HLA-DPB1, HLA-DRB5	Antipsychotic
**Filgotinib**	JAK1, TYK2	JAK inhibitor, Anti-inflammatory
**Floxacillin**	HLA-B, HLA-DQB1, HLA-DRA	Antibiotic
**Lumiracoxib**	HLA-DQA1, HLA-DQB1	NSAID
**Tofacitinib**	JAK1, TYK2	JAK inhibitor
Aldesleukin	IL2RA, CD28, SOCS1, STAT4	Recombinant analog of IL-2
Arsenic trioxide	MAPK1, MAPK3	Chemotherapeutic
Baminercept	LTBR, TNFSF14	Antirheumatic
Belatacept	CD86, CD28	Selective costimulation modulator,
		Antirheumatic, Immunosuppressant
Briakinumab	IL12B, IL12A	Anti-IL-12 antibody
Fostamatinib	JAK1, CAMK2G, LCK	Spleen tyrosine kinase inhibitor
Leucovorin	GATA3, RUNX3	Folic acid analogs
Mercaptopurine	GATA3, HLA-DQA1	Antineoplastic
Methotrexate	SLAMF1, TNFAIP3	Antineoplastic
Purvalanol	MAPK1, MAPK3	MAPK1, MAPK3 inhibitor
Ruxolitinib	JAK1, IL7R	JAK1/2 inhibitor, Antineoplastic
Seliciclib	MAPK1, MAPK3	CDK inhibitor
Ulixertinib	MAPK1, MAPK3	ATP-competitive ERK1/2 inhibitor
Ustekinumab	IL12B, IL12A, TNFAIP3	Anti-IL12/IL23 antibody

#### 3.3.2 Drugs Targeting Common Risk Pathways/Differentially Expressed Genes Based on Transcriptome Data

Drugs targeting common risk pathways were searched using CTD, and 435 drugs were found for 10 common risk pathways, of which no drug targeted more than or equal to three common risk pathways. In addition, 415 drugs targeting common DEGs were obtained using DrugBank and DGI databases, of which 12 drugs targeted more than or equal to two common DEGs, most of which were antineoplastic and immunodepressant drugs. There were three drugs targeting hub common DEGs including cisplatin, doxorubicin, and vincristine (**Table 5**).

**Table 5:** Drugs targeting more than or equal to two common DEGs. Black-bordered letters represent drugs targeting more than or equal to two hub DEGs.

Drug	Target gene	Effect
**Cisplatin**	PIK3CA, STAT1	Antineoplastic
**Doxorubicin**	CYP1B1, GATA3, NT5E, PIK3CA	Antineoplastic
**Vincristine**	ABCB4, GATA3, PIK3CA	Antineoplastic
Benzquinamide	ABCB4, CHRM3	Antihistaminic, Anticholinergic
Curcumin	ABCB4, CAMK2A	Antibacterial, anti-inflammatory, hypoglycemic,
		antioxidant, wound-healing, and antimicrobial activities
Celecoxib	ALOX12, PIK3CA	NSAID
Cyclophosphamide	CYP1B1, GATA3	Antineoplastic, Immunosuppressant
Docetaxel	CYP1B1, PIK3CA	Antihistaminic
Fluorouracil	CYP1B1, PIK3CA	Antihistaminic
Letrozole	CYP1B1, PIK3CA	Aromatase inhibitor
Paclitaxel	CYP1B1, PIK3CA	Antihistaminic
Cetuximab	NT5E, PIK3CA	Antihistaminic

#### 3.3.3 Comparison of Candidate Drugs Identified From GWAS Data and Transcriptome Data

**Figure 6:**
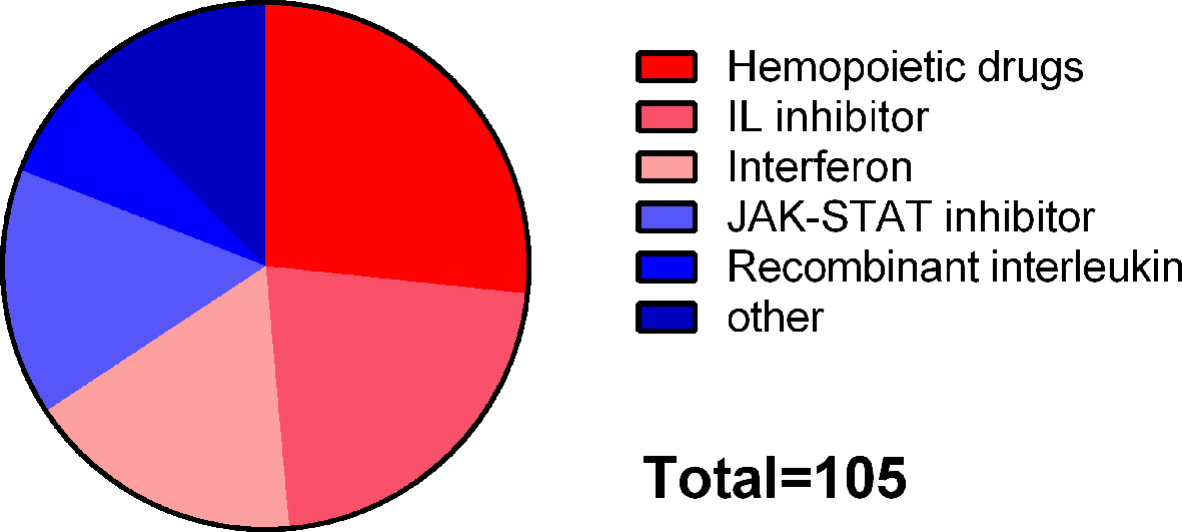
Classification of drugs targeting both common susceptibility pathways and common risk pathways.

“JAK-STAT signaling pathway” and “influenza A” were included in common susceptibility pathways and common risk pathways at the same time. There were 295 drugs targeting common susceptibility pathways and 435 drugs targeting common risk pathways, with 105 overlaps that were mainly divided into 6 categories: hematopoietic drugs (26.7%), interleukin (IL) inhibitor (21.9%), interferon (INF) (17.1%), JAK-STAT inhibitor (15.2%), and recombinant IL (6.7%) (**Figure 6**). There were 168 drugs targeting common susceptibility genes and 415 drugs targeting common DEGs with 28 overlaps, of which 17 were antineoplastic drugs, such as cyclophosphamide, mercaptopurine, and thioguanine (**Supplementary Table S7**).

#### 3.3.4 Comparison of Target Genes of Approved/Investigational Drugs

Sixteen approved drugs with 39 target genes and 44 investigational drugs with 88 target genes of MS as well as 5 approved drugs with 9 target genes and 30 investigational drugs with 44 target genes of SS were obtained in this study (**Supplementary Table S8**). Through comparing hub common susceptibility genes and target genes of available drugs, we found that *IL2RA* and *HLA-DRB1* that were identified as hub common susceptibility genes were the targets of daclizumab and glatiramer acetate, respectively, which were approved drugs of MS. Besides, hub common susceptibility genes *JAK1* and *TYK2* were the targets of filgotinib and tofacitinib, which were under investigation for SS treatment (**Table 6**). There was no intersection between target genes of MS investigational drugs/SS-approved drugs and hub common susceptibility genes.

**Table 6:** Approved/investigational drugs targeting hub common susceptibility genes.

Drug	Target gene	Effect
Glatiramer acetate	HLA-DRB1	Immunomodulator
Daclizumab	IL2RA	Anti-CD25(IL-2) antibody
Filgotinib	JAK1, TYK2	JAK inhibitor
Tofacitinib	JAK1, TYK2	JAK inhibitor

## 4 Discussion

We observed the comorbidity of MS with SS or specific antibodies of SS in clinical work. Combining with studies showing their similarities in environmental factors and pathogenesis, we speculated that MS and SS may have common genetic susceptibility factors and molecular mechanisms, which lack relevant reports. In this study, we analyzed their common genes and pathways based on GWAS data and transcriptome data and identified candidate drugs targeting common pathways and genes. In addition, a comparison between target genes of approved/investigational drugs and common susceptibility genes was performed to explore whether the drugs of MS and SS can provide reference for each other.

Through the analysis of GWAS data, 28 common susceptibility pathways of MS and SS were obtained, in which immune system-related pathways such as the “Th1, Th2 and Th17 cell differentiation” accounted for 21.43%, indicating that T cells played an important role in the pathogenesis of MS and SS. GO analysis of common susceptibility genes showed that the most significant BP was the “interferon-gamma-mediated signaling pathway”, which also supported the vital contribution of T cells. Because we all know that INF-γ is the main pro-inflammatory factor of T cells. Th1 and Th17 cells were activated and enhanced in the brain tissue, cerebrospinal fluid, and peripheral blood of MS patients, of which INF-γ and interleukin-17 (IL-17) played a vital role in the pathogenesis of MS as their main cytokines (19). CD4^+^ T cells also made a valuable contribution to SS, especially in the early periods, as it was reported that it accounted for more than 75% of the infiltration around the salivary gland (SG) epithelial cells of SS patients (20). INF-γ that is secreted by Th1 cells upregulated the expression of *major histocompatibility complex II (MHC II)* and *CXC motif chemokine receptor 3 (CXCR3) ligands* on the surface of epithelial cells, resulting in more recruitment of pathogenic T cells to the lesions and aggravation of inflammation. Besides, highly activated Th17 cells in the peripheral blood, salivary gland, and labial gland were observed in SS patients (21). Our study found that pathways related to infectious diseases accounted for 32.14% of the common susceptibility pathways, indicating that MS and SS both associated with infections such as EBV and human T-cell leukemia virus 1 (HTLV1), which may be the initiating factors of autoimmune responses in genetically susceptible individuals. EBV is one of the most studied viruses. Infectious mononucleosis caused by EBV infection doubled the risk of MS and increased Epstein–Barr nuclear antigen 1 (EBNA1) antibody in serum, and EBV DNA and antibodies in brain tissue were also found in MS patients (22). At the same time, the increased EBV DNA in salivary gland tissue and anti-EBV antibodies in serum were also found in SS patients. Besides, there was a relationship between the serological characteristics of previous EBV infection and anti-SSA and anti-SSB antibodies (23). Some studies showed that EBV infection of B cells was related to MS, while infection of epithelial cells was related to SS (24, 25). In addition, pathways related to autoimmune diseases also accounted for 32.14% of the common susceptibility pathways, suggesting that genetic and environmental factors were common factors in most autoimmune diseases.

Ten common risk pathways of MS and SS were obtained by the analysis of the transcriptome data. In particular, “JAK-STAT signaling pathway” and “influenza A” were included in both common susceptibility pathways and common risk pathways at the same time, which may participate in the pathogenesis of both MS and SS and strongly contribute to their shared mechanism. Abnormal activation of JAK-STAT signaling pathway had been observed in many autoimmune diseases, such as rheumatoid arthritis (RA), systemic lupus erythematosus (SLE), psoriatic arthritis, and psoriasis. It was a pathway that mediated the signal transduction activated by a variety of cytokines, resulting in cell activation, proliferation, and the release of inflammatory factors, and also contributed primarily to the differentiation and function of Th1 and Th17 cells. Four JAKs (*JAK1*, *JAK2*, *JAK3*, and *TYK2*) and 7 STATs (*STAT1*, *STAT2*, *STAT3*, *STAT4*, *STAT5A*, *STAT5B*, and *STAT6*) had been identified in mammals, which could specifically combine with each other to transduce a unique signal (26). *JAK1* and *STAT3* were the most significant hub common susceptibility genes in this study, *TYK2* was also one of the hub common susceptibility genes, and *STAT1* was one of the hub common DEGs. Enhanced activation of *STAT1* and *STAT3* in peripheral blood lymphocytes was observed in MS patients, indicating the activation of the pro-inflammatory response (27, 28). Moreover, highly activated *STAT3* in T cells of clinically isolated syndrome (CIS) patients can predict the possibility of progression to MS (29). In addition, elevated *STAT3* in brain myeloid cells was observed in MS patients and experimental autoimmune encephalomyelitis (EAE) model, proving its vital contribution to the regulation of myeloid cell function (30, 31). Moreover, selective ablation of *STAT3* made mice resistant to EAE induction by inhibiting the production of pathogenic T cells (31). Immunohistochemical analysis of labial salivary gland (LSG) in SS patients showed that the expression of *JAK1* and *JAK2* was enhanced in ductal cells and acinar cells, respectively. The expression of *STAT1* was increased in salivary gland tissues of SS patients (32). Gene expression profile analysis of epithelial cells from SS patients’ LSG also proved the significant enrichment of JAK-STAT-related genes (33), which could reduce the expression of *autophagy-related gene-5 (ATG5)* in LSG epithelial cells and increase the expression of pro-inflammatory factors such as IL-6 (34).

Fourteen hub common susceptibility genes were obtained by constructing pathway–gene network and PPI network. Eight of them belonged to *human leukocyte antigen (HLA) class Ⅱ* genes. *HLA* genes code for the *HLA* molecules that are the glycoproteins located on the surface of antigen-presenting cells and participate in immune response by recognizing and presenting exogenous (I) and endogenous (II) antigens. Variations of *HLA* genes contribute to genetic susceptibility of MS about 20%–60% (35). In particular, *HLA-DRB1* had the strongest association with MS, which was consistent with the results of our study. Structural study showed that HLA-DRB1*1501 affected the peptide binding site of the *HLA* molecule and promoted combination with autoantigens like myelin basic protein, resulting in 3-fold increased risk of MS (36). The gene locus with the strongest association with SS was also located in *HLA* genes. However, due to racial differences, the current conclusions were not consistent. A meta-analysis integrating 23 studies showed that HLA-DRB1*03:01, DQA1*05:01, and DQB1*02:01 were all associated with the risk of SS (37). Lessard et al. (38) found that *HLA-DQB1* was mostly related to primary SS in the European population, followed by *HLA-DQA1*. Li et al. (39) found that *HLA-DRB1/HLA-DQA1* and *HLA-DPB1* had the strongest association in the Chinese Han population. *HLA-DRB1*, *HLA-DQB1*, *HLA-DQA1*, and *HLA-DPB1* were all included in the hub common susceptibility genes in our study. *JAK1* and *STAT3* were the most significant in non-*HLA* genes, which was consistent with our conclusion of pathway enrichment. In addition, Mendelian randomization study showed that there was a causal relationship between the genetic susceptibility of *IL2RA* and MS, whose targeted drug daclizumab had distinct efficacy in reducing gadolinium-enhanced lesions and annual recurrence rate (40). The level of serum soluble *IL2RA* in SS patients increased significantly, which was closely related to clinical characteristics such as erythrocyte sedimentation rate (ESR) and C-reactive protein (CRP) (41). Studies have found that microglia with overactivated *MAPK* can injure oligodendrocytes (OLs) and lead to demyelination, while *MAPK* inhibitors can promote the differentiation of OL precursor cells into mature OLs and myelination, which may become a new method for the treatment of demyelinating diseases (42). MAPK/ERK pathway is a strong stimulator for cell survival, growth, and proliferation with an important effect on anti-apoptosis, which can be affected by JAK-STAT signal pathway at the same time. Activation of MAPK/ERK pathway was also found in peripheral blood and LG cells of SS patients, and *MAPK* inhibitors could significantly reduce the production of IL-17-related pro-inflammatory factors (43). At present, polymorphism of *JAK1*, *STAT3*, *IL2RA*, *MAPK1*, and *TYK2* related to SS is not yet reported, which may become the candidate genes for SS in the future.

Through constructing a PPI network, 3 hub common DEGs were obtained, including *STAT1*, *GATA3*, and *PIK3CA*. *GATA3* is a transcription factor required for Th2 cell differentiation, which promotes the development of Th2 cells and prevents the proliferation of Th1 cells. Studies had reported that the expression of *GATA3* in peripheral blood of MS patients decreased, while the increase of its expression reduced the severity of EAE (44, 45). *PI3K* is widely expressed and participates in multiple inflammatory responses. Inhibition of *PI3K* may inhibit activated Th1/Th17 cells and microglia (46). Moreover, animal experiments showed that *PI3K* inhibitor could reduce the condition of EAE and SS (46, 47).

As for treatment, glucocorticoid is mainly used for acute attack of MS, and disease-modifying therapy (DMT) is mainly used for remission, including INF-β, glatiramer acetate, teriflunomide, dimethyl fumarate, sphingosine-1-phosphate (SP1) receptor regulator, monoclonal antibody, and chemotherapeutic drugs. In terms of treatment for SS, topical treatment is first considered, including muscarinic agonists and artificial saliva for dryness of mouth and artificial tears for dryness of eyes. In addition, glucocorticoids and cyclosporine A are considered in serious conditions. Glucocorticoids, immunosuppressants, and biological agents can be used when the system involvement appears, but they are all empirical except hydroxychloroquine.

In order to identify candidate drugs for MS or SS, we searched for the drugs targeting common pathways and genes based on GWAS data and transcriptome data, respectively. As for common pathways, we found that there were 105 overlaps between agents from GWAS and transcriptome data, of which 60.9% were immunomodulatory drugs, including IL inhibitors, JAK-STAT inhibitors, recombinant IL, and INF, suggesting their potential therapeutic effects on MS and SS, especially on their comorbidity. Besides, as for common genes, 28 overlaps between agents from GWAS and transcriptome data were obtained, among which methotrexate, cyclophosphamide, and mercaptopurine were used for SS according to clinical experience and for MS when first-line and second-line drugs have no effect. In addition, we compared the target genes of approved/investigational drugs for MS and SS with common susceptibility genes and found that the target genes of glatiramer acetate and daclizumab were *HLA-DRB1* and *IL2RA*, respectively. Glatiramer acetate is a first-line treatment for MS, as a myelin basic protein (MBP) analog that specifically competes with myelin antigen to bind T cells. Therefore, it may have a specific immunosuppressive effect in MS. Although it can also induce Th2 cells and inhibit dendritic cells and monocytes, the immunomodulatory effect in SS needs to be further studied. Daclizumab was also used for MS but was delisted in 2018 due to its side effects of severe encephalitis and meningitis, resulting in no consideration to be used for SS. However, the possibility of *IL2RA*-targeted drugs in the treatment of MS and SS cannot be ignored.

Combining the results of identification of drugs targeting common pathways/genes and analysis of target genes of approved/investigational drugs, we suggested that JAK-STAT inhibitors may be potential common therapeutic drugs for MS and SS. At present, *JAK* inhibitors have been applied for the treatment of several autoimmune diseases, including tofacitinib, baricitinib, and upadacitinib, which have been approved by the Food and Drug Administration (FDA) and European Medicines Agency (EMA) for the treatment of RA, and tofacitinib is also used for psoriatic arthritis (48). Previous studies have shown that *JAK* inhibitors play a therapeutic role in MS and SS mice. The inhibitors of *JAK1/2* such as baricitinib, ruxolitinib, and AZD148 can attenuate the severity of EAE by inhibiting Th1 and Th17 cells and inducing regulatory T (Treg) cells (49–51). Tofacitinib is a nonselective *JAK* inhibitor (*JAK1*, *JAK2*, *JAK3*, *TYK2*) and has a beneficial effect on myelination by inhibiting the inflammatory cascade (52). In a phase 2 clinical trial, the topical application of tofacitinib reduced the infiltration of inflammatory cells and expression of pro-inflammatory factors (tumor necrosis factor -α, IL-23, and IL-17A) in cornea and attenuated the dryness of eyes (53). Filgotinib is a *JAK1* inhibitor that is indicated for the treatment of active moderate to severe rheumatoid arthritis alone or in combination with methotrexate. After treatment with filgotinib, significant increase in salivary secretion and decrease in lymphocyte infiltration were observed in SS mice (54). The clinical trials of filgotinib (NCT03100942) and baricitinib (NCT04916756) for SS are underway, and the results are expecting. In addition, *STAT* inhibitors (such as Statatic) was found to attenuate the symptoms and inflammatory indicators of EAE. Therefore, JAK-STAT inhibitors may have therapeutic effects on patients with MS or SS. In particular, JAK-STAT inhibitors used for MS/SS may also contribute to the therapy of SS/MS and may also become a choice for the comorbidity of MS and SS. However, there is a case report of reversible multifocal CNS demyelination in a patient with seropositive RA during tofacitinib therapy (55). So, it is worthwhile to further study the role of JAK-STAT pathway in MS and therapeutic effect of JAK-STAT inhibitors in MS.

This study had some limitations, as follows. First, although GWAS can identify multiple SNPs related to diseases, it only can reveal a small part of genetic factors related to complex diseases, and there existed racial heterogeneity. However, GWAS is one of the main methods to find genetic variations of complex diseases at present. Second, only two GWAS reports were available, leading to a small number of SS susceptibility genes. Therefore, when analyzing GWAS data, we first performed the pathway enrichment of disease susceptibility genes and then obtained the MS or SS susceptibility genes on the common susceptibility pathways, which were defined as the common susceptibility genes in this study. Third, the cutoff for DEGs was relatively low in our study (*P*-value <0.05 and fold change ≥1.2). When we increased the fold change to 1.3 as used for common cases and made subsequent analysis, the common DEGs of MS and SS decreased to 40 including 3 DEGs in CD4^+^ T cells and 37 DEGs in B cells. The detailed results of fold change ≥1.3 and comparison with the results of fold change ≥1.2 were shown in **Supplementary Table S9**. GO annotation showed that the most significant BP was “positive regulation of mast cell degranulation”; the only significant CC was “cytoplasm,” and the only significant MF was “double-stranded DNA binding” (**Supplementary Figure S1**). The PPI network with 11 nodes and 8 edges was constructed, and one hub common DEG (*STAT1*) with degree ≥4 was identified (**Supplementary Figure S2**). In terms of KEGG pathway enrichment, 24 and 43 significant risk pathways of MS and SS as well as 6 common risk pathways were obtained (**Supplementary Table S10**). Although the cutoff for DEGs was relatively low, the results based on transcriptome data were meaningful to a certain extent, especially combined with the results based on GWAS data.

## 5 Conclusion

Our study revealed the shared mechanism of MS and SS based on the available GWAS data and transcriptome data. Seventeen genes such as *HLA-DRB1*, *JAK1*, *STAT3*, and *STAT1* and JAK-STAT signal pathway have been identified as key genes and pathway in the shared mechanism, which may be the genetic and molecular bases of clinical comorbidity of MS with SS. Importantly, JAK-STAT inhibitors may become potential therapeutic drugs for MS and SS, especially for their comorbidity.

## Data Availability Statement

The datasets presented in this study can be found in online repositories. The names of the repository/repositories and accession number(s) can be found in the article/**Supplementary Material**.

## Author Contributions

XXH, XW, XMR, TTZ, and JF: conceptualization. XXH, XW, XYY, and JF: data curation. XXH, XMR, RW, TTZ, and JF: formal analysis. XXH, XMZ, RW, and JF: funding acquisition. XXH: writing—original draft. XXH, DW, TTZ, and JF: writing—review and editing. All authors read and approved the final article.

## Funding

This work was supported by the project of Nature Scientific Foundation of Heilongjiang Province (ZD2020H004) and Postgraduate Research & Practice Innovation Program of Harbin Medical University (YJSCX2020-98HYD).

## Conflict of Interest

The authors declare that the research was conducted in the absence of any commercial or financial relationships that could be construed as a potential conflict of interest.

## Publisher’s Note

All claims expressed in this article are solely those of the authors and do not necessarily represent those of their affiliated organizations, or those of the publisher, the editors and the reviewers. Any product that may be evaluated in this article, or claim that may be made by its manufacturer, is not guaranteed or endorsed by the publisher.

## Supplementary Material

Supplementary Figure S1 GO terms in the biological process, cellular component, and molecular function category of common DEGs (Fold change ≥ 1.3).

Supplementary Figure S2 Protein-protein interaction network of common DEGs (Fold change ≥ 1.3). Nodes represent DEGs, edges represent combined score of PPI. The higher the combined score, the thicker the edge.
