# Supplementary figures and images for: The Shared Mechanism and Candidate Drugs of Multiple Sclerosis and Sjögren’s Syndrome Analyzed by Bioinformatics Based on GWAS and Transcriptome Data
Source: Front Immunol. 2022 Mar 9;13:857014. doi: 10.3389/fimmu.2022.857014 (PMC8959321; doi:10.3389/fimmu.2022.857014)

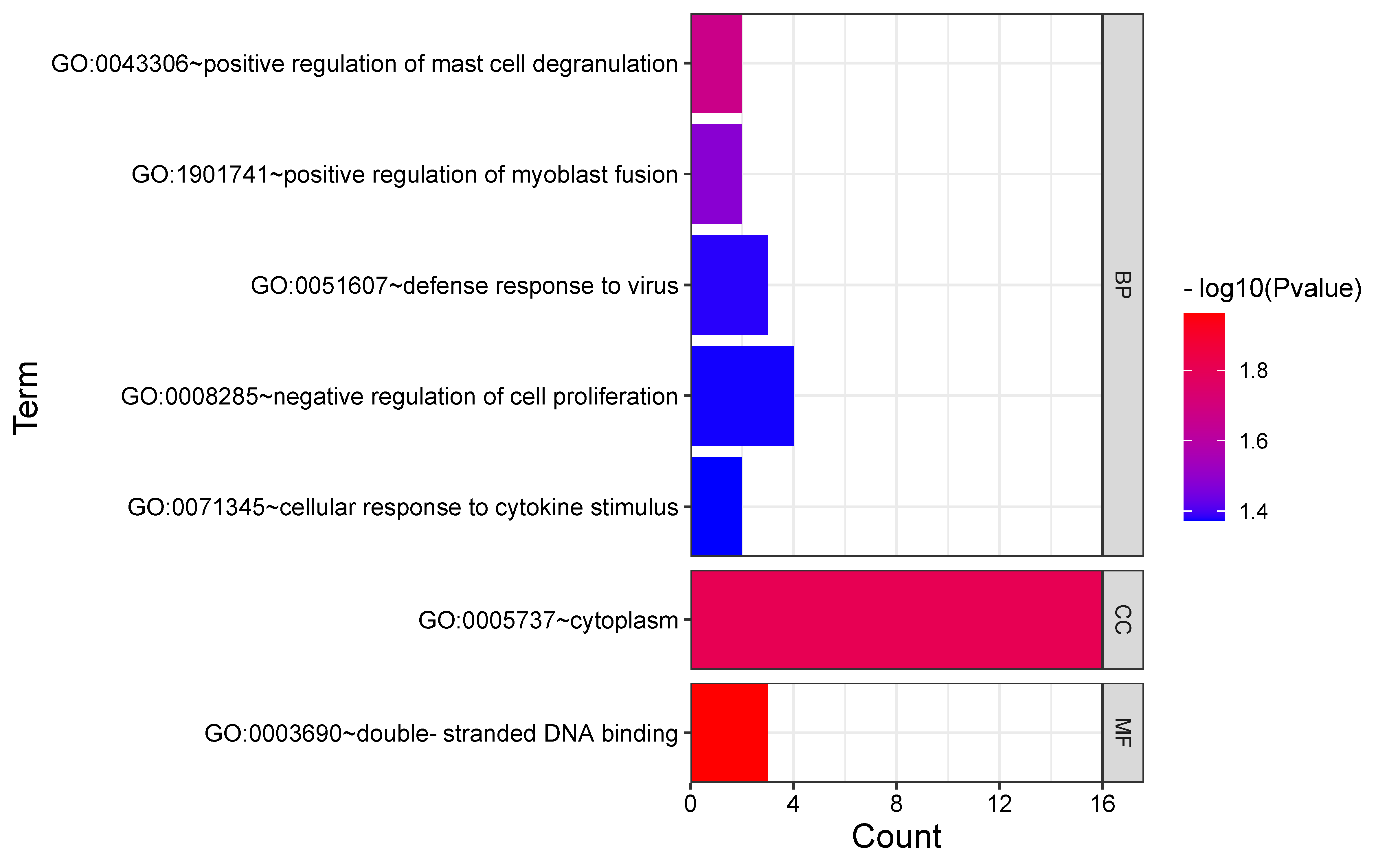

Supplement: Supplementary Figure S1 — GO terms in the biological process, cellular component, and molecular function category of common DEGs (Fold change ≥ 1.3). [file Image_1.tiff]
